# CD74-Targeted Cathepsin-Inhibitor Antibody–Drug Conjugate Triggers Apoptosis in DLBCL

**DOI:** 10.3390/cells15030291

**Published:** 2026-02-04

**Authors:** Ihab Abd-Elrahman, Noha Khairi, Reut Sinai-Turyansky, Ivan Zlotber, Riki Perlman, Emmanuelle Merquiol, Galia Blum, Dina Ben Yehuda

**Affiliations:** 1 Department of Hematology, Hadassah Medical Center, Jerusalem 9112001, Israel; ihabae@savion.huji.ac.il (I.A.-E.);; 2 Faculty of Medicine, The Hebrew University of Jerusalem, Jerusalem 9112001, Israel

**Keywords:** diffuse large B-cell lymphoma, cathepsins, antibody–drug conjugates (ADC), cell death

## Abstract

Transcriptomic analyses of public datasets (TCGA and GTEx) revealed that both *CD74* and *Cathepsin L *(CTSL) are significantly overexpressed in diffuse large B-cell lymphoma (DLBCL) compared to normal tissues, and that their expression levels are highly correlated to each other (Spearman R = 0.64, *p* = 3 × 10^−46^). Kaplan–Meier analysis showed that elevated expression of both genes is associated with reduced overall survival (OS), defining a high-risk *CD74*+/*CTSL*+ DLBCL subgroup. This is the first study demonstrating coordinated overexpression of CD74 and CTSL and proposing their dual targeting via antibody–drug conjugates (ADCs) to improve outcomes in relapsed or refractory DLBCL. Cysteine cathepsins, a family of proteases, are upregulated in many cancers, facilitating tumor invasion and metastasis. Cathepsins are overexpressed and play key roles in DLBCL progression. GB111-NH_2_, a potent broad-spectrum cathepsin inhibitor, significantly reduced cathepsin activity in lymphoma cell lines and patient samples. GB111-NH_2_ treatment increased apoptosis and caspase-3 activation in DLBCL patient cells and chronic lymphocytic leukemia (CLL) mononuclear cells. Here, we developed a modified cathepsin inhibitor, M-GB, containing a maleimide linker for site-specific antibody conjugation. While M-GB alone has poor cell permeability, when conjugated to an antibody, it forms an ADC (M-GB–ADC) that selectively induces lymphoma cell death. One M-GB–ADC demonstrated high specificity for CD74-expressing lymphoma cells while exhibiting minimal toxicity to non-target cells in vitro. Our findings highlight the potential of another M-GB–ADC as a targeted therapy for overcoming rituximab resistance and treatment failure in DLBCL. This strategy enhances therapeutic efficacy and represents a preclinical proof-of-concept treatment option by directing a cathepsin-inhibitor payload specifically to malignant B cells.

## 1. Introduction

Diffuse large B-cell lymphoma (DLBCL) is the most common aggressive lymphoma, accounting for roughly 25–40% of Non-Hodgkin Lymphoma (NHL) diagnoses [1]. Durable remission can often be achieved with first-line chemoimmunotherapy (e.g., R-CHOP). However, ~30% of patients do not respond to initial therapy or eventually relapse with refractory disease [2,3,4]. Notably, trials intensifying or modifying R-CHOP have not significantly improved outcomes for this high-risk group [5]. Moreover, DLBCL is a heterogeneous disease comprising multiple molecular subtypes [6], and individuals with aggressive phenotypes (e.g., activated B-cell subtype) have especially poor prognoses [5]. In recent years, new therapies have expanded the treatment landscape [7]. For example, adding the anti-CD79B ADC polatuzumab vedotin to frontline therapy yielded a modest progression-free survival (PFS) benefit (a 2-year PFS of ~77% vs. 70% with R-CHOP alone) [8]. Likewise, chimeric antigen receptor (CAR) T-cell therapies targeting CD19 can induce durable remissions in ~40–50% of heavily pretreated DLBCL patients. Bispecific T-cell engager antibodies (e.g., glofitamab) have shown high response rates in refractory DLBCL (ORR ~52%, CR ~39%) [9] and gained regulatory approvals. Still, a substantial fraction of patients does not respond to these newer immunotherapies or eventually relapses, underscoring a continued need for innovative targeted treatments, particularly for those who do not benefit from the current treatments [10]. Cysteine cathepsins, a family of lysosomal proteases, have emerged as important mediators of cancer progression and potential therapeutic targets [11]. These enzymes (including cathepsins B, L and S) are often overexpressed in malignancies and contribute to extracellular matrix degradation [12], tumor invasion, and metastasis [13]. Cathepsins can also modulate the tumor microenvironment; for instance, excessive cathepsin activity in the tumor niche degrades antigenic proteins and immune receptors, impairing anti-tumor immunity [14]. Cathepsin L (CTSL) is dysregulated in various cancers and has been implicated in cancer cell growth, angiogenesis, metastasis, and the development of treatment resistance [13]. In DLBCL, cathepsin S is known to facilitate antigen presentation and support tumor cell survival (through invariant chain processing), and elevated cathepsin S expression is generally associated with more aggressive disease [15]. Broad-spectrum cysteine cathepsin inhibitors can induce apoptotic death of leukemia and lymphoma cells, highlighting the vulnerability of these tumors to cathepsin blockade [16]. However, no cathepsin-directed agents have yet reached clinical approval, likely due in part to concerns about off-target effects in normal tissues [14]. A targeted delivery strategy is therefore attractive to maximize tumor-specific cathepsin inhibition while minimizing systemic toxicity.

We chose to focus on CD74, the invariant chain of MHC class II, as a cell surface “anchor” for delivering a cathepsin inhibitor to lymphoma cells. When expressed on the plasma membrane, invariant chain is known as CD74, and it is highly expressed in many B-cell malignancies. Over 85% of non-Hodgkin lymphoma cases (and cell lines) express CD74 at high levels, as do the majority of chronic lymphocytic leukemias and multiple myeloma cells [17]. Importantly, CD74 undergoes extremely rapid internalization upon antibody binding, at a rate of ~8 × 10^6^ molecules internalized per cell per day [18]. This efficient endocytic trafficking makes CD74 an attractive target for antibody-based therapy, as an ADC can be rapidly shuttled inside the cell [5]. Indeed, the biology of CD74 provides a strong rationale for ADC development: in vivo, CD74 normally functions as an MHC II chaperone and is cleaved by proteases (e.g., cathepsin S) during antigen presentation [15], and it also serves as a receptor for the pro-inflammatory cytokine MIF, triggering survival signaling pathways [18]. By targeting CD74, one can exploit its high tumor expression and quick internalization to deliver therapeutic payloads directly into malignant B cells.

CD74 has emerged as a promising therapeutic target in hematologic malignancies, and early clinical studies support this strategy. To date, only one CD74-directed agent has been reported in clinical trials: the humanized anti-CD74 monoclonal antibody milatuzumab. In a phase I/II study in relapsed/refractory NHL, milatuzumab (combined with anti-CD20 antibody veltuzumab) produced a modest overall response rate of 24% [5]. This limited efficacy of the naked antibody suggested that a more potent approach—such as an ADC—would be needed to fully exploit CD74. ADC development for CD74 is underway: for example, STRO-001 is a novel anti-CD74 ADC bearing a non-cleavable maytansinoid toxin (DAR = 2) that has demonstrated potent preclinical activity. STRO-001 was cytotoxic at low-nanomolar concentrations in 88% of tested lymphoma and myeloma cell lines, and induced tumor regressions and cures in DLBCL and mantle cell lymphoma xenograft models [5]. These findings have led to an ongoing first-in-human trial of STRO-001 in B-cell malignancies. Notably, STRO-001 and other CD74-targeted ADCs in development employ conventional cytotoxic payloads (e.g., microtubule inhibitors), which can diffuse to bystander cells and damage normal proliferating cells [19]. There remains a substantial need for innovative ADC design by exploring non-traditional payloads with different mechanisms of action.

Here, we propose a dual-targeting therapeutic strategy that is, to our knowledge, the first of its kind in lymphoma: an anti-CD74 ADC that delivers a cytotoxic cathepsin inhibitor payload. This approach is based on our observation that a subset of DLBCL tumors co-overexpress CD74 and CTSL, a co-expression signature associated with significantly worse patient survival. We hypothesized that simultaneously targeting a cell-surface antigen (CD74) and an intracellular pro-tumor enzyme (CTSL) could provide a two-pronged attack on DLBCL cells, particularly in relapsed or refractory cases. By conjugating a cathepsin inhibitor to an anti-CD74 antibody, we aim to achieve precision delivery of the drug. The inhibitor remains essentially inert until the ADC binds to CD74 and is internalized into the lymphoma cell. This design minimizes exposure of healthy cells to the payload and could confer a wider therapeutic window than traditional ADCs. In summary, the rationale for dual targeting CD74 and CTSL in DLBCL is to exploit a tumor-specific vulnerability (CD74^high^/CTSL^high^ lymphomas) with a novel ADC that can overcome resistance mechanisms and spare normal tissues. In the following section, we present the development and characterization of a CD74-directed CTSL inhibitor ADC and evaluate its mechanistic feasibility as a targeted strategy to overcome current DLBCL treatment challenges.

## 2. Materials and Methods

Cell lines and culture: DLBCL cell lines OCI-Ly19 and OCI-Ly3 (kindly provided by Dr. Neta Goldschmidt) were used, as well as SU-DHL-6 (obtained from ATCC). Additional hematologic cancer lines included acute myeloid leukemia HL-60 (from ATCC). Burkitt’s lymphoma cell line B-Blast (provided by Hanna Ben-Bassat, Hadassah Medical Center) was also included. OCI-Ly19 cells were grown in Iscove’s Modified Dulbecco’s Medium (IMDM); OCI-Ly3 and all other cell lines were maintained in RPMI-1640. All media were supplemented with 10% fetal calf serum, 100 U/mL penicillin, 100 μg/mL streptomycin, and 1 mM L-glutamine, and cells were cultured at 37 °C in a 5% CO_2_ humidified incubator.

Gene expression analysis: Gene expression data for CD74 and CTSL were obtained from the GEPIA2 web platform (http://gepia2.cancer-pku.cn), which integrates RNA-seq expression profiles from The Cancer Genome Atlas (TCGA) and Genotype-Tissue Expression (GTEx) projects. Expression values were reported in transcripts per million (TPM). For tissue-wide analysis, bar and dot plots were generated in GEPIA2, comparing tumor tissues (from TCGA) to corresponding normal tissues (from TCGA or GTEx). Correlation analysis: To assess co-expression between CTSL and CD74, Spearman correlation coefficients were calculated using GEPIA2 on DLBCL (DLBC) and Acute myeloid leukemia (LAML) tumor data, normalized to CD19 gene expression. Gene expression values were log_2-transformed TPM. Survival analysis: Kaplan–Meier curves were generated via GEPIA2 to evaluate the impact of CD74 or CTSL expression on patient OS. Patients were stratified into High- and Low-expression groups based on median expression. Differences in survival were evaluated by log-rank test, and hazard ratios (HR) with 95% confidence intervals were reported from the GEPIA2 output. Gene expression and survival analyses were conducted using bulk RNA-seq data integrated from TCGA and GTEx projects. These analyses represent population-level expression and were not adjusted for individual clinical prognostic factors such as IPI, cell-of-origin, or TP53 status.

Patient samples: With approval from the Hadassah Medical Center IRB (Helsinki Committee #HMO-0144-16) and written informed consent, primary samples were collected from patients and healthy donors. Peripheral blood mononuclear cells (PBMCs) (at least three biological repeats), were isolated by Ficoll-Paque density gradient centrifugation from the blood of patients with Chronic Lymphocytic Leukemia (CLL), Diffuse Large B-Cell Lymphoma (DLBCL), and Marginal Zone Lymphoma (MZL), as well as from healthy donors. To ensure physiological relevance and assess drug activity in the presence of circulating enzymes, CLL cells were cultured in medium supplemented with autologous serum (collected from the same patient) 24 h prior to and during the assay. All patient samples were obtained at the time of diagnosis (prior to therapy) and used fresh in culture to maintain the native stability and metabolic profile of the primary cells.

Cathepsin activity assays: To measure cysteine cathepsin activity, detergent lysates were prepared from OCI-Ly19 and OCI-Ly3 cells, as well as from patient and healthy donor PBMCs. Samples were pre-treated with either DMSO (vehicle control) or 5 µM GB111-NH_2_ (broad cathepsin inhibitor [20]) for 30 min at room temperature, followed by the addition of 1 µM of the fluorescent activity-based probe GB123 [21]. After 1 h of labeling at 37 °C, equal amounts of protein from each sample were separated by SDS-PAGE, and active cathepsins were visualized by scanning the gels for fluorescence (Typhoon 9410 imager (GE Healthcare, Piscataway, NJ, USA)). In some experiments, purified recombinant human CTSL or cathepsin S enzymes were incubated in acetate buffer (pH 5.5) with various concentrations of inhibitors (GB111-NH_2_ or M-GB) or 0.1% DMSO for 30 min, followed by labeling with 1 µM GB123 for 30 min. These samples were analyzed by SDS-PAGE and fluorescent scanning as above. In some experiments (where indicated), intact cells were treated with GB111-NH2 followed by GB123 cathepsin probe, then cells were lysed, separated by SDS PAGE that were scanned for fluorescence.

Cathepsin immunoprecipitation: To confirm the identities of active cathepsins in cell lysates, OCI-Ly19 lysates labeled with GB123 (as described above) were subjected to immunoprecipitation with cathepsin-specific antibodies. Anti-CTSL or anti-cathepsin S antibodies (Abcam) were added to cleared lysates, followed by protein A/G agarose beads (Santa Cruz Biotechnology, Dallas, TX, USA). After incubation and washing, bound proteins were eluted in SDS sample buffer at 100 °C for 10 min. The fluorescently labeled supernatant (unbound fraction) and immunoprecipitated eluate were analyzed by SDS-PAGE and gel scanning. A ~20 kDa band corresponding to CTSL and a ~25 kDa band corresponding to cathepsin S were thus isolated and identified.

Caspase-3 activity and apoptosis assays: OCI-Ly19 cells were treated with 5 µM GB111-NH_2_ for 1 h and then returned to fresh medium. After 24 h, caspase-3 activity was measured in cell lysates (100 µg protein) using a colorimetric substrate (Ac-DEVD-pNA; (CaspACE assay system Assay, Promega, Madison, WI, USA). Cleavage of this substrate by active caspase-3 releases p-nitroaniline, which was quantified by its absorbance at 405 nm as an indicator of caspase activity. In parallel, cells were stained with Annexin V–FITC and analyzed by flow cytometry to assess phosphatidylserine exposure (an early apoptosis marker). For CLL patients’ samples, mononuclear cells were similarly treated with 5 µM GB111-NH_2_ for 1 h and then assessed 24 h later by Annexin V staining and flow cytometry.

NF-κB reporter assay: OCI-Ly19 cells were stably transfected with an NF-κB response element-driven NanoLuc luciferase reporter (NlucP/NF-κB-RE vector, Promega). Transfected cells were treated for 24 h under various conditions: with or without lipopolysaccharide (LPS, 0.5 µg/mL) to stimulate NF-κB, and in the presence or absence of 5 µM GB111-NH_2_ or 10 µM SC-514 (a selective NF-κB p65 inhibitor, Calbiochem, San Diego, CA, USA). After treatments, NanoLuc luciferase activity in cell lysates was measured using a plate reader to quantify NF-κB transcriptional activity.

Flow cytometry for cell death and surface markers: To evaluate cell death under different treatments, OCI-Ly19 cells were exposed to various agents for 24 h as follows: (i) 5 µM GB111-NH_2_ for 1 h (followed by washout and an additional 23 h in fresh medium), (ii) an unconjugated control antibody (10 µg/mL IgG), (iii) 2.5 µg/mL etoposide (a DNA-damaging chemotherapeutic), or (iv) an antibody–M-GB conjugate (ADC) generated as described below. After treatment, cells were stained with propidium iodide (PI) and analyzed by flow cytometry to quantify membrane impermeability (PI uptake), an indicator of late-stage cell death.

For cell surface CD74 detection, OCI-Ly19 cells were treated for 24 h ± 0.5 µg/mL LPS and ±5 µM GB111-NH_2_. Cells were then stained on ice with FITC-conjugated anti-CD74 antibody (BD Biosciences, Franklin Lakes, NJ, USA) and analyzed by flow cytometry; mean fluorescence intensity was compared between conditions.

ADC preparation (M-GB conjugation): M-GB is a modified derivative of GB111-NH_2_ containing a maleimide functional group in place of the benzyloxycarbonyl moiety. This design enables conjugation to antibodies via reaction with surface-accessible cysteine residues. To prepare antibody–M-GB conjugates, the antibody of interest (0.3 mg/mL, e.g., rituximab or an anti-CD74 mAb) was incubated with M-GB (5 µM) for 30 min at 37 °C with gentle agitation. The maleimide on M-GB reacts spontaneously with thiol groups on the antibody, forming a stable thioether bond. The reaction mixture was then concentrated and washed with a 10 kDa molecular weight cutoff centrifugal filter (Vivaspin, GE, Goettingen, Germany) to remove unbound M-GB and other small molecules, yielding purified M-GB–ADC.

Statistical analysis: Data were analyzed using GraphPad Prism software (Version 10). Results are presented as mean ± standard deviation (SD) of at least three independent biological replicates. Prior to significance testing, the distributional properties of each dataset were evaluated to ensure the assumptions of parametric testing were satisfied. For comparisons between two groups, statistical significance was determined using two-tailed unpaired Student’s *t*-tests. When group variances were unequal, an un-pooled variance *t*-test was used to maintain statistical stringency. *p*-values < 0.05 were considered statistically significant.

## 3. Results

**Figure 1:**
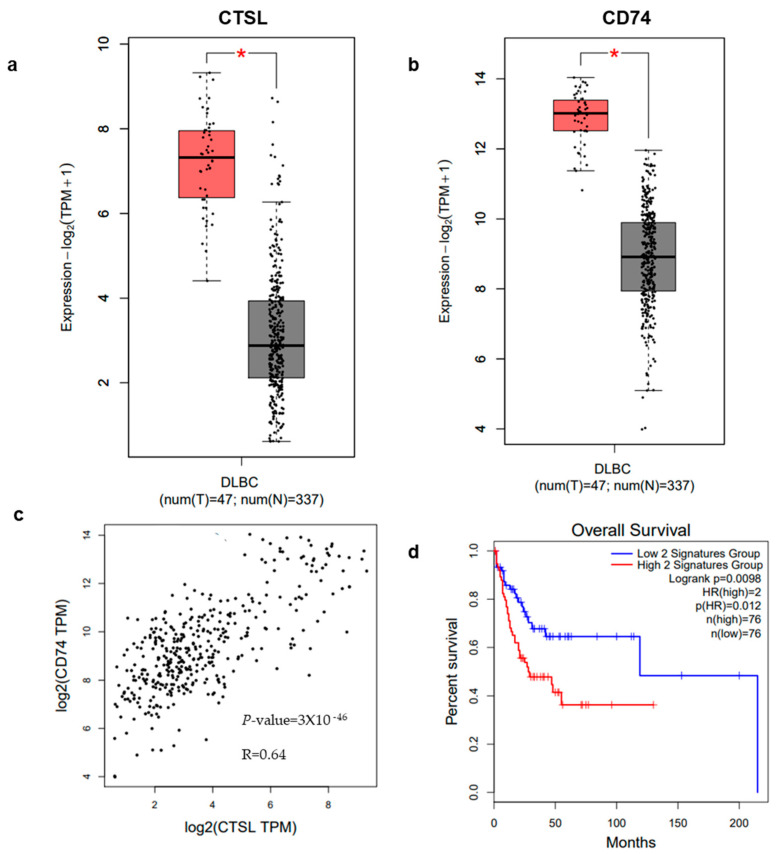
Expression, correlation, and prognostic value of CTSL and CD74 across cancers. CTSL (**a**) and CD74 (**b**) expression levels in DLBCL are presented as red bar plots with individual data points, demonstrating significant elevation compared to normal lymphatic tissues (gray bar). (**c**) Correlation analysis between CTSL and CD74 expression in DLBCL reveals a good correlation (Spearman R = 0.64, *p* = 3 × 10^−46^). (**d**) Kaplan–Meier survival plot of Overall Survival (OS) in DLBCL patients stratified by CD74/CTSL co-expression (normalized to CD19). Patients with high expression (red line, *n* = 76) show significantly reduced survival compared to the low expression group (blue line, *n* = 76) (Log-rank *p* = 0.0098; Hazard Ratio [HR] = 2). Data are presented as mean ± SEM. The red asterisk (*) in a and b indicates a statistically significant difference between the two groups (*p* < 0.05).

CD74 and CTSL are co-overexpressed in hematologic malignancies and are associated with poor prognosis: To explore the clinical significance of these markers, we analyzed gene expression datasets from TCGA and GTEx. Both CTSL (Figure 1a) and CD74 (Figure 1b) are markedly overexpressed in DLBCL compared to normal tissues. Correlation analysis (Figure 1c) revealed a good correlation between CTSL and CD74 expression (Spearman R = 0.64, *p* = 3 × 10^−46^), suggesting transcriptional co-regulation. Importantly, survival analysis confirmed that this signature has prognostic value. Patients were stratified into high- and low-expression groups based on a combined gene signature (CD74 and CTSL normalized to CD19). As shown in Figure 1d, patients with concurrent high expression of these genes exhibited significantly reduced Overall Survival (OS) compared to the low-expression group (Log-rank *p* = 0.0098). The analysis yielded a Hazard Ratio (HR) of 2, indicating that patients with high CD74/CTSL co-expression have a two-fold increased risk of mortality, supporting the potential utility of this signature as a negative prognostic marker.

**Figure 2:**
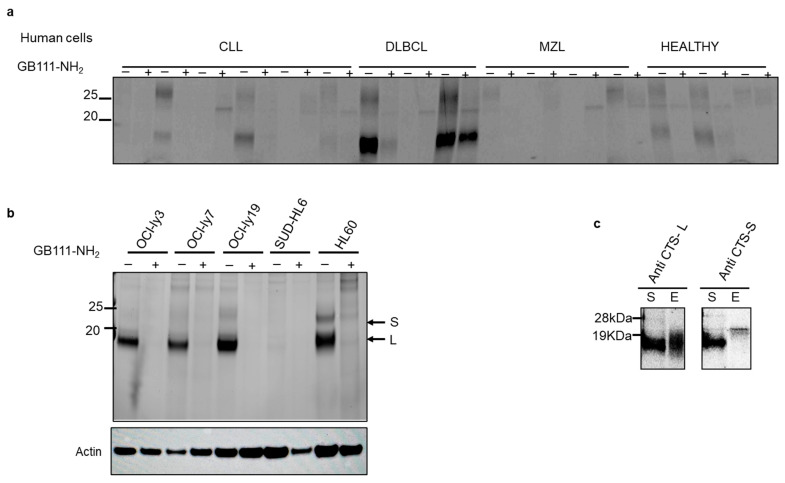
Cathepsin activities in hematological malignancy patient cells and cell lines. (**a**) Lysates of cells from healthy donors and patients with hematologic malignancies (CLL, DLBCL, MZL) were pretreated with DMSO or with a cathepsin inhibitor (5 µM GB111-NH_2_) for 30 min, then labeled with 1 µM GB123 for 1 h. Equal amounts of protein were loaded for SDS-PAGE, and active cathepsins were visualized by fluorescent gel scanning. Each pair of lanes (vehicle and inhibitor pretreated) was from a different patient. (**b**) Cathepsin activities in cell lines of various hematologic malignancies. Cell lysates were pretreated with 5 µM GB111-NH_2_ or DMSO vehicle for 20 min and then labeled with 1 µM GB123 for an hour. Labeled lysates were analyzed by SDS-PAGE and fluorescent scanning. Arrows indicate bands corresponding to active cathepsins. (**c**) Immunoprecipitation of active cathepsins from OCI-Ly19 cells. Lysates were labeled with GB123 as in (**a**), then immunoprecipitated with antibodies against CTS-L left, or cathepsin S (CTS-S), right. The fluorescent signals in the supernatant (S) and immunoprecipitated eluate (e) fractions were analyzed by SDS-PAGE and scanning, confirming the identities of the ~20 kDa (CTSL) and ~25 kDa (cathepsin S) bands. Due to limited sample availability, loading controls were not performed for this panel; results are presented as qualitative validation of the quantitative trends observed in cell lines.

Cathepsin activity is elevated in lymphoma patient samples and cell lines: We next examined cysteine cathepsin enzymatic activity in primary lymphoma cells and cell lines. Peripheral blood mononuclear cells from lymphoma patients (collected at diagnosis, before therapy) were compared to healthy donor cells. Using the fluorescent probe GB123, which irreversibly labels active cathepsins [21], we observed elevated cathepsin B, L and S activities in lysates from CLL and DLBCL patients’ cells relative to healthy controls (Figure 2a). In biological replicate #3 (DLBCL), shown in Figure 2a, while the reduction in cathepsin activity following GB111-NH_2_ treatment was not as substantial as in other samples, a noteworthy inhibitory effect was observed. Similarly, high cathepsin activity was detected in hematologic cancer cell lines, including DLBCL (OCI-Ly7, OCI-Ly19, SU-DHL-6) and AML (HL-60) (Figure 2b). Multiple fluorescent bands corresponding to active cathepsins were observed on SDS-PAGE gels. To identify these bands, we immunoprecipitated GB123-labeled OCI-Ly19 lysates with cathepsin S- or CTSL-specific antibodies. The results showed that the ~20 kDa band represents CTSL and the ~25 kDa band represents cathepsin S (Figure 2c). DLBCL and AML cell lines showed prominent bands at ~20 and ~25 kDa (CTSL and cathepsin S, respectively). Among the DLBCL lines, OCI-Ly7 and OCI-Ly19 had higher CTSL activity than SU-DHL-6. We also noted a weaker band around ~32 kDa—likely cathepsin B—present in several cell lines and especially intense in HL-60 cells (Figure 2b). As expected, pretreatment with the inhibitor GB111-NH_2_ greatly reduced all cathepsin activity signals, confirming that the GB123 probe specifically reports on cysteine cathepsin activity that GB111-NH_2_ effectively blocks.

**Figure 3:**
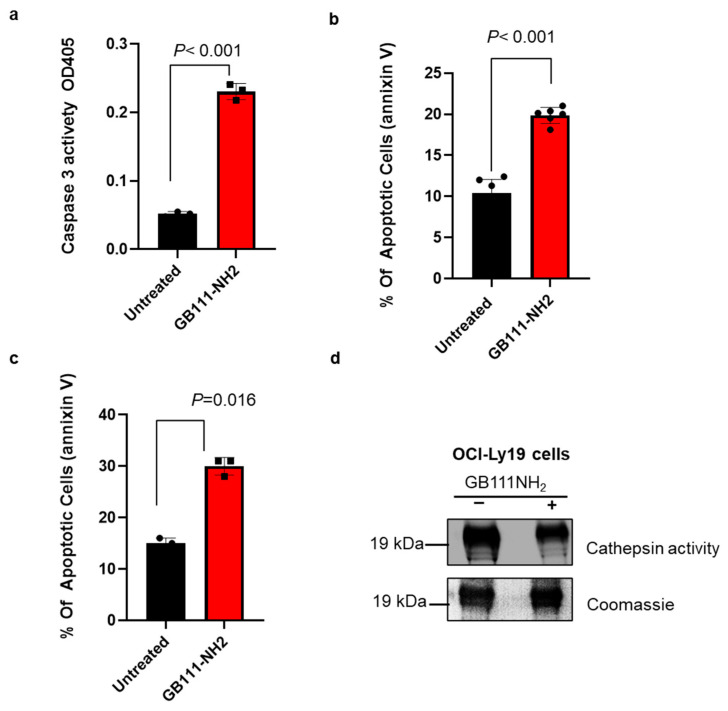
Cathepsin inhibition induces apoptosis. (**a**) OCI-Ly19 cells were treated with 5 µM GB111-NH_2_ for 1 h. Caspase-3 activity was measured 24 h later in cell lysates (100 µg protein) using a colorimetric substrate (Ac-DEVD-pNA), which produces a 405 nm signal upon cleavage. (**b**) OCI-Ly19 cells were treated with 5 µM GB111-NH_2_ for 1 h, then 24 h later, they were stained with Annexin V and analyzed by flow cytometry to assess apoptosis. (**c**) Mononuclear cells from CLL patients (*n* = 3) were treated with 5 µM GB111-NH_2_ for 1 h, stained with Annexin V 24 h later, and analyzed by flow cytometry. (**d**) Lysates from the CLL cells in (**c**) were labeled with 1 µM GB123 for 1 h and analyzed as in Figure 1a; the gel was then Coomassie-stained to verify equal loading. In (**a**–**d**), graphs represent mean ± SD (*n* = 4). *p* < 0.05 (two-tailed Student’s *t*-test) for inhibitor-treated vs. control. Symbols represent individual replicates (**a**,**b**) or samples (**c**).

Cathepsin inhibition induces apoptosis in lymphoma cells: Given the elevated cathepsin activity in lymphoma, we investigated whether blocking cathepsin activity induces cell death. OCI-Ly19 DLBCL cells were treated with 5 µM GB111-NH_2_ for an hour, and apoptosis was assessed 24 h later. GB111-NH_2_ treatment led to a significant increase in apoptosis, as evidenced by a ~4.5-fold rise in caspase-3 activity (Figure 3a) and a ~2.5-fold increase in Annexin V-positive cells (Figure 3b) compared to untreated controls. Similarly, inhibiting cathepsins with GB111-NH_2_ triggered apoptosis in primary cells from CLL patients: CLL mononuclear cells (*n* = 3) showed increased Annexin V staining after 5 µM GB111-NH_2_ treatment (Figure 3c, *p* = 0.016). In these patient CLL cells, cathepsin S activity was initially high and was reduced by ~40% following GB111-NH_2_ treatment (Figure 3d).

**Figure 4:**
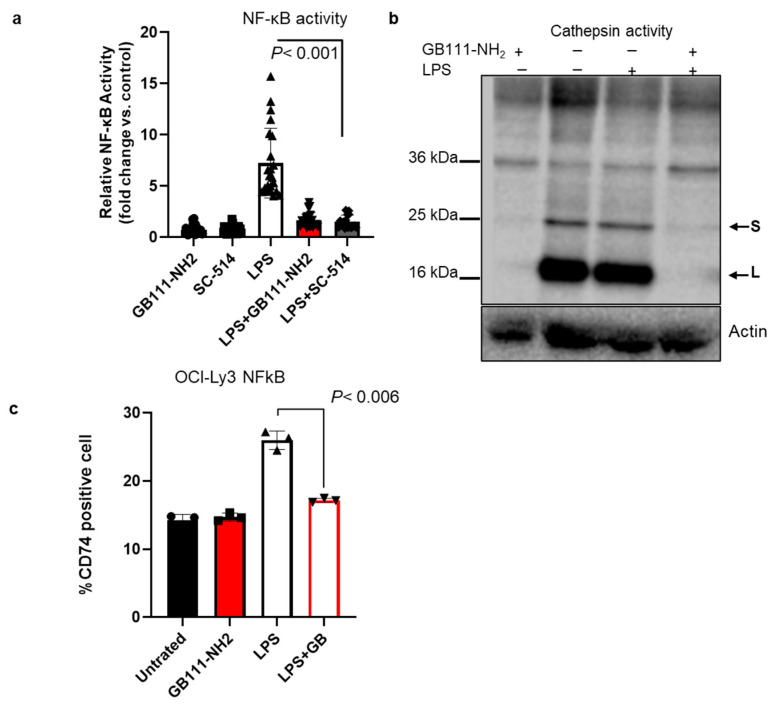
Cathepsin inhibition negatively regulates NF-κB and CD74. (**a**) OCI-Ly19 cells stably transfected with an NF-κB luciferase reporter were treated for 24 h ± LPS (0.5 µg/mL) in the presence or absence of 5 µM GB111-NH_2_ or 10 µM SC-514 (a p65 inhibitor). NF-κB activity was measured by luciferase assay. LPS stimulation increased NF-κB activity (relative light units, RLUs) substantially, and GB111-NH_2_ suppressed this induction, similar to the effect of SC-514 (*n* = 22). (**b**) Cell lysates from the experiment in (**a**) were labeled with 1 µM GB123 for 1 h. SDS-PAGE and fluorescent scanning analyzed equal protein samples to assess active cathepsins. GB111-NH_2_ effectively blocked the cathepsin activity bands under both basal and LPS-stimulated conditions. (**c**) Cell surface CD74 expression was measured by flow cytometry in OCI-Ly19 cells treated for 24 h with GB111-NH_2_ (5 µM), LPS (0.5 µg/mL), or LPS + GB111-NH_2_. LPS increased surface CD74, whereas GB111-NH_2_ reduced surface CD74 levels (especially when combined with LPS). Graphs show mean ± SD (*n* = 6–10). *p* < 0.05 for indicated comparisons (Student’s *t*-test). Symbols represent individual replicates.

Cathepsin inhibition suppresses NF-κB activation and CD74 upregulation: Because CD74 ICD-mediated signaling is linked to NF-κB p65 activation, we analyzed whether cathepsin inhibition affects NF-κB activity and CD74 levels. OCI-Ly19 cells stably expressing an NF-κB luciferase reporter were treated with LPS to stimulate NF-κB. LPS stimulation increased NF-κB-driven luciferase activity by ~7-fold (Figure 4a). Notably, the addition of GB111-NH_2_ markedly suppressed this LPS-induced NF-κB activation, reducing it to near-baseline levels (comparable to the effect of the direct p65 inhibitor SC-514; Figure 4a). In parallel, GB111-NH_2_ effectively blocked cathepsin activity in these cells under both basal and LPS-stimulated conditions (Figure 4b). We also examined cell surface CD74 expression by flow cytometry. LPS treatment increased CD74 surface levels, whereas GB111-NH_2_ abrogated this LPS-induced upregulation of CD74 (Figure 4c). Thus, cathepsin activity facilitates NF-κB activation and CD74 expression in lymphoma cells, and its inhibition can attenuate these pro-survival signals.

**Figure 5:**
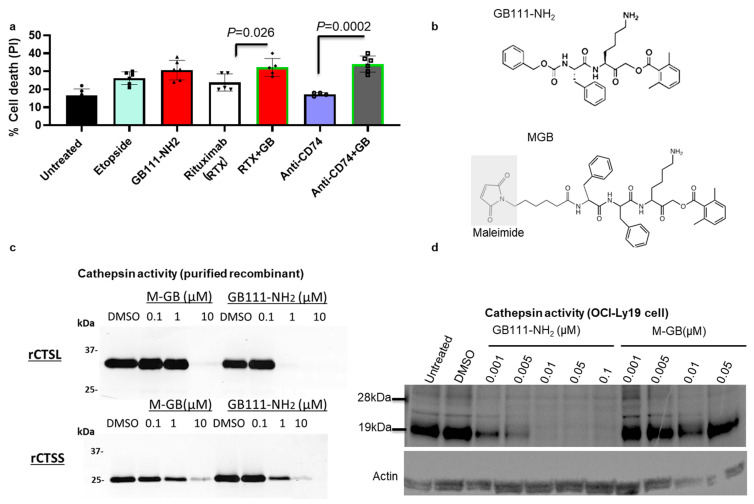
Design and effects of a cathepsin inhibitor ADC. (**a**) OCI-Ly19 cells were treated under different conditions for 24 h: 5 µM GB111-NH_2_ (1 h pretreatment, then removal) with or without an antibody treatment (anti-CD74 or Rituximab), 2.5 µg/mL etoposide (24 h), for 24 h. Cells were then stained with PI and analyzed by flow cytometry for apoptosis (*n* = 5–6). GB111-NH_2_, in combination with the antibody or etoposide, increased cell death compared to the antibody or etoposide alone. (**b**) Chemical structures of the parent inhibitor GB111-NH_2_ and the maleimide-containing derivative M-GB. The maleimide linker (gray box) replaces the benzyloxycarbonyl group, enabling conjugation to antibodies. (**c**) Inhibition of purified recombinant cathepsins L (rCTSL) and S (rCTSS) by GB111-NH_2_ vs. M-GB. Recombinant enzymes (pH 5.5) were incubated with the indicated concentrations of each inhibitor or with 0.1% DMSO (vehicle) for 30 min, followed by labeling with 1 µM GB123 for 30 min. SDS-PAGE analyzed samples, and cathepsin activity was visualized by fluorescent scanning. (**d**) Inhibition of endogenous cathepsin activity in intact OCI-Ly19 cells. Cells were treated with 5 µM GB111-NH_2_ or 5 µM M-GB for 2 h, then labeled with 1 µM GB123 for 1 h. SDS-PAGE and scanning analyzed cathepsin activity in cell lysates. GB111-NH_2_ strongly reduced intracellular cathepsin signals, whereas M-GB had a minimal effect. Graphs show mean ± SD (*n* = 5). *p* < 0.05 (two-tailed *t*-test). Symbols represent individual replicates.

Combining cathepsin inhibition with other therapies enhances lymphoma cell killing: Given the pro-apoptotic effect of cathepsin inhibition alone, we next tested whether combining GB111-NH_2_ with existing lymphoma therapies could improve tumor cell killing. OCI-Ly19 cells were treated for 24 h with 10 µg/mL of an antibody (either rituximab, an anti-CD20 monoclonal antibody, or an anti-CD74 monoclonal antibody) in the presence or absence of 5 µM GB111-NH_2_ (GB111-NH_2_ was added 1 h before antibody addition and present during the antibody treatment). In both cases, with anti-CD20 or anti-CD74, the addition of GB111-NH_2_ significantly increased cell death compared to treatment with the antibody alone (Figure 5a). This suggests that cathepsin inhibition can potentiate the cytotoxic effects of other lymphoma therapies. However, since GB111-NH_2_ is a membrane-permeable inhibitor that could also enter and affect normal cells, a more targeted delivery method is desirable to achieve cancer cell-specific cathepsin inhibition.

Design of a cathepsin inhibitor ADC (M-GB) and its properties: We therefore designed a tumor-targeted cathepsin inhibitor by modifying GB111-NH_2_ into a form suitable for antibody conjugation. The resulting inhibitor, M-GB, replaces the benzyloxycarbonyl group of GB111-NH_2_ with a maleimide linker moiety (Figure 5b, maleimide highlighted in gray), enabling attachment to antibody cysteine residues via thioether bond formation. Importantly, we expected that M-GB would reduce cell permeability (due to the maleimide) and thus would primarily be active only when delivered inside target cells via an antibody vehicle. Peptide mapping analysis, performed at the Mass Spectrometry Unit of The Hebrew University of Jerusalem (Supplementary Table S1), revealed that M-GB conjugation occurred at multiple cysteine residues distributed across the antibody structure (Supplementary Figure S1). Modified peptides carrying the characteristic M-GB mass addition (+736–737 Da) were identified with high confidence (AScore = 1000) in three distinct regions. First, conjugation was observed at the interchain cysteine sites within the Fc constant region (residues ~254–278, peptide D.TLMISR…), consistent with solvent-accessible thiols. Second, significant conjugation was detected within the Variable Heavy (VH) domain (residues ~75–96, peptide K.SSSTAY…), indicating modification of intrachain cysteines. Third, modifications were identified in the C-terminal region (residues ~555–580, peptide R.VEAEDA…). The high ion intensity observed across these distinct sites confirms that the conjugation process was not limited to a single region but involved both interchain and intrachain cysteine residues. Based on this high ion intensity and the identification of modifications at these three distinct regions, we provide a semi-quantitative estimate of the average DAR as greater than 2. We acknowledge that this estimate is derived from peptide mapping rather than intact-mass LC-MS. There was no evidence of off-target conjugation to lysine residues or N-terminal amines.

We first confirmed that the structural changes in M-GB did not abolish its ability to inhibit cathepsin activity. In a biochemical assay, recombinant human CTSL or CTSS were incubated with increasing concentrations of M-GB or GB111-NH_2_, followed by the addition of the fluorescent cathepsin probe GB123 to measure remaining enzyme activity. M-GB proved to be a potent inhibitor of both CTSL and cathepsin S (Figure 5c and Supplementary Figure S1a). Inhibition of CTSL required higher M-GB concentrations compared to GB111-NH_2_, whereas cathepsin S was inhibited similarly by both compounds (Figure 5c). We next tested the ability of M-GB to inhibit cathepsins within intact cells. OCI-Ly19 cells were treated with either M-GB or GB111-NH_2_ for 24 h, then labeled with GB123 to visualize residual cathepsin activity. As expected, GB111-NH_2_ strongly inhibited intracellular cathepsin activity (Figure 5d), while M-GB, in contrast, had minimal effect on intracellular cathepsins (Figure 5d). These results confirm that M-GB retains cathepsin-inhibitory potency, but on its own, it does not readily penetrate cells, supporting its use as an ADC payload to achieve cell-specific cathepsin inhibition.

**Figure 6:**
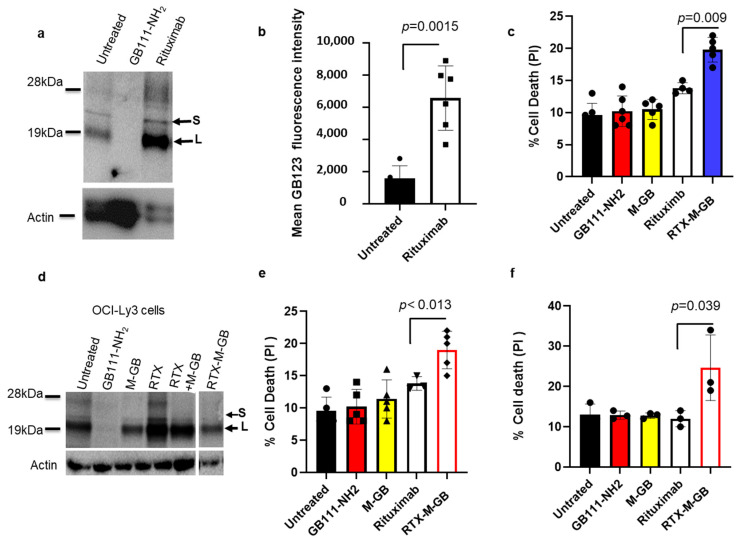
Rituximab–M-GB conjugate counters rituximab-induced cathepsin activity and enhances cell death. (**a**) OCI-Ly3 DLBCL cells were treated with or without rituximab (anti-CD20, 10 µg/mL) for 24 h. Cell lysates were labeled with 1 µM GB123 for 1 h, separated by SDS-PAGE, and scanned for active cathepsins (representative gel is shown). (**b**) Fluorescent quantification of cathepsin band intensities from (**a**). Rituximab treatment increased cathepsin activity bands (~25 kDa) by ~3.8-fold compared to untreated cells (*n* = 6). (**c**) OCI-Ly3 cells were treated for 24 h with rituximab alone, M-GB alone, or a rituximab–M-GB conjugate (RTX-M-GB). Cells (*n* = 6) were stained with PI and analyzed by flow cytometry for cell death. The RTX-M-GB conjugate induced higher cell death than rituximab or M-GB alone. (**d**) After the treatments in (**c**), cell lysates were labeled with 1 µM GB123 for 1 h and analyzed by SDS-PAGE and scanning. Rituximab alone increased cathepsin activity (as in (**a**)), whereas RTX-M-GB treatment prevented this increase, keeping cathepsin activity low. (**e**,**f**) Mononuclear cells from a CLL patient (*n* = 5) (**e**) and a MZL patient (*n* = 3) (**f**) were isolated by Ficoll. Cells were treated as indicated for 24 h, stained with PI and analyzed by flow cytometry. RTX-M-GB treatment resulted in greater cell death (PI-positive cells) than either rituximab or M-GB alone in these primary samples. Graphs show mean ± SD. *p* < 0.05 for R-M-GB vs. rituximab (*t*-test). Symbols represent individual replicates.

Rituximab conjugated with M-GB overcomes therapy-induced cathepsin activity and kills lymphoma cells: Prior studies have shown that cathepsin levels can increase in tumor cells following certain treatments, potentially contributing to therapy resistance. In our experiments, we observed that treating OCI-Ly3 DLBCL cells with rituximab (anti-CD20) led to a ~3.8-fold increase in intracellular cathepsin activity compared to untreated cells (Figure 6a,b; *p* = 0.0015). To counteract this effect, we conjugated M-GB to rituximab, thereby delivering the inhibitor specifically to CD20-positive lymphoma cells. The conjugate (designated RTX-M-GB) was prepared by mixing the inhibitor with the antibody as described in the Methods Section (Section 2). OCI-Ly3 cells were then treated for 24 h with rituximab alone, M-GB alone, or the RTX-M-GB conjugate. Treatment with RTX-M-GB resulted in an increase in cell death (PI uptake) compared to rituximab alone (*p* = 0.049; Figure 6c), suggesting enhanced cytotoxic potential. Moreover, cells receiving RTX-M-GB did not show the enhanced cathepsin activity that was observed with rituximab-only treatment; instead, their cathepsin activity remained low, comparable to that of untreated cells (Figure 6d and Supplementary Figure S1a). Adding rituximab and M-GB, which are not bound and thus M-GB cannot enter the cells, does not inhibit cathepsin activity. Thus, coupling a cathepsin inhibitor to rituximab not only enhances tumor cell killing but also prevents the therapy-induced rise in cathepsin activity that might otherwise limit rituximab’s effectiveness.

To validate the clinical relevance of these findings, we evaluated the efficacy of the conjugate using primary patient-derived B-cell malignancies. Peripheral blood mononuclear cells (PBMCs) were isolated from patients with Chronic Lymphocytic Leukemia (CLL) (Figure 6e) and Marginal Zone Lymphoma (MZL) (Figure 6f). Consistent with our cell line data, treatment with RTX-M-GB resulted in significantly greater cell death than Rituximab alone in CLL patients’ cells (*p* = 0.013, *n* = 5). A similar, notable trend toward enhanced cell death was observed in the MZL patients’ cells (*p* = 0.039, *n* = 3). Furthermore, the conjugate demonstrated robust cytotoxicity against primary CLL cells cultured in autologous serum. This indicates that the linker–payload remains stable and functionally active in a human blood environment rich in proteases and esterases, supporting its potential for clinical stability. In contrast, the unconjugated inhibitor controls (GB111-NH2 and M-GB) induced minimal toxicity, confirming that the enhanced potency relies on specific, antibody-mediated delivery.

**Figure 7:**
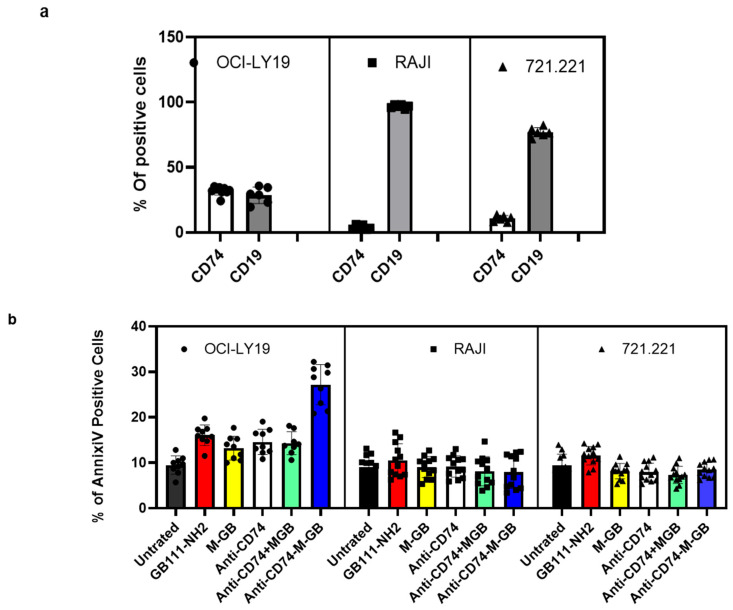
CD74-targeted M-GB ADC selectively kills CD74-positive cells. (**a**) Flow cytometry analysis of CD74 and CD19 surface expression on OCI-Ly19 (DLBCL), Raji (Burkitt lymphoma), and 721.221 (lymphoblastoid) cells. OCI-Ly19 cells express high levels of CD74 and CD19, whereas Raji and 721.221 cells have low CD74 expression. (**b**) Cells were treated with an anti-CD74–M-GB conjugate (CD74–M-GB) or control conditions for 24 h. Apoptosis was measured by Annexin V staining and flow cytometry. CD74–M-GB induced significant apoptosis in OCI-Ly19 cells, while Raji and 721.221 cells were largely unaffected. Graphs represent mean ± SD (*n* = 12). *p* < 0.05 for OCI-Ly19 vs. Raji or 721.221 (two-tailed *t*-test). Symbols represent individual replicates.

CD74-targeted M-GB ADC selectively kills CD74-positive lymphoma cells: Patients with relapsed or refractory DLBCL often develop resistance to rituximab, in part due to loss or downregulation of CD20. One strategy to overcome this is to target an alternative antigen that remains highly expressed in B-cell malignancies even when CD20 is low, such as CD74 [16]. We therefore generated an anti-CD74 ADC by conjugating M-GB to a chimeric anti-CD74 monoclonal antibody (LL1 clone). To test the specificity and efficacy of this conjugate (CD74–M-GB), we compared its effects on cells with high vs. low CD74 expression. OCI-Ly19 cells express high levels of CD74 (as well as the B-cell marker CD19), whereas Raji (Burkitt lymphoma) and 721.221 (B-lymphoblastoid) cells have very low CD74 surface expression (Figure 7a). The cells were treated with CD74–M-GB or control conditions for 24 h, then analyzed for apoptosis by Annexin V staining. CD74–M-GB induced significantly higher apoptosis in OCI-Ly19 cells compared to either Raji or 721.221 cells (Figure 7b). Specifically, OCI-Ly19 showed a marked increase in Annexin V-positive cells, whereas Raji and 721.221 exhibited minimal cell death in response to the ADC. These differences were highly significant (*p* = 3.08 × 10^−11^ for OCI-Ly19 vs. Raji; *p* = 1.15 × 10^−9^ for OCI-Ly19 vs. 721.221). Treatment with the anti-CD74-M-GB ADC resulted in a significant reduction in cathepsin activity (see Supplementary Figure S1b,c). These results indicate that the CD74–M-GB ADC selectively targets and kills CD74-expressing lymphoma cells while sparing CD74-negative cells, highlighting its potential as a therapeutic agent for CD74-positive malignancies.

## 4. Discussion

Non-Hodgkin lymphoma (NHL) remains a major global health concern, with ~560,000 new cases and ~270,000 deaths worldwide in 2023 (Globocan 2023). Diffuse large B-cell lymphoma (DLBCL), the most aggressive NHL subtype, often presents advanced-stage disease and extranodal involvement. Despite improvements in survival with R-CHOP chemotherapy, a significant subset of patients, particularly those with high-risk features such as refractory disease or “double-hit” genetics, require more effective frontline and salvage therapies. New immunotherapies, including CAR T cells and bispecific antibodies, have shown promise, but at least half of patients with relapsed DLBCL still fail to achieve durable remission with these approaches [22]. This persistent unmet need underscores the importance of developing novel targeted strategies, particularly for patients who relapse after or are resistant to rituximab-based therapy.

In this study, we identified CD74 and CTSL as co-expressed drivers of aggressive DLBCL and demonstrated that their dual targeting reveals prognostic and therapeutic vulnerabilities. Transcriptomic analyses of patient datasets defined a high-risk CD74^+^/CTSL^+^ gene expression signature associated with inferior outcomes. Using GEPIA2, we found that concurrent high expression of these genes was associated with significantly poorer overall survival in DLBCL and AML (Figure 1d). While these findings suggest that elevated CD74/CTSL co-expression may mark an aggressive subset of lymphoid malignancies, we emphasize that this analysis is an exploration and requires validation in independent datasets. These observations align with emerging research implicating cysteine cathepsins as facilitators of tumor progression and therapy resistance [23]. CTSL has been linked to cancer cell invasion and metastasis in solid tumors and to treatment resistance mechanisms in hematologic malignancies [23]. By showing that cathepsin inhibition induces apoptosis in DLBCL cells, including primary patient samples treated with the broad-spectrum inhibitor GB111-NH_2_, we provide evidence that cathepsin activity is a functional contributor to tumor survival that can be therapeutically exploited [24].

A key finding of our work is that dual targeting of CD74 and CTSL can overcome established mechanisms of treatment resistance. Rituximab (anti-CD20) remains a mainstay of DLBCL therapy, yet many patients relapse, often with loss or downregulation of CD20 on tumor cells [25]. We observed that rituximab treatment alone induced a compensatory increase in cathepsin activity, potentially reflecting an adaptive survival mechanism [26,27,28]. In contrast, our [Rituximab]-GB111-NH_2_ conjugate effectively countered this upregulation. A critical factor in ADC design is the internalization rate of the target antigen. Unlike antigens such as CD74, which undergoes rapid receptor-mediated endocytosis, CD20 exhibits high surface stability and slower internalization kinetics. However, our functional data (Figure 6) confirms that the conjugate overcomes this barrier. The conjugate successfully reaches the lysosomal compartment, as evidenced by the direct inhibition of intracellular cathepsin processing. This demonstrates that despite the inherently slower kinetics of CD20, the internalization rate of the conjugate is sufficient to deliver a pharmacologically active concentration of the inhibitor.

Beyond rescuing Rituximab sensitivity, our data support the direct targeting of CD74 as a primary therapeutic strategy. CD74 is consistently expressed on B-cell tumors even when CD20 is lost [18], offering a vital alternative for escape variants. Previous clinical experience with the anti-CD74 antibody milatuzumab (24% response rate) suggested that CD74 targeting required greater potency to achieve durable responses [5], a concept further validated by the preclinical success of STRO-001, which showed that antibody–drug conjugation can transform modestly active antibodies into highly potent therapeutics [5]. Consistent with these findings, our CD74-directed ADC achieved potent, selective cytotoxicity in lymphoma cells. Importantly, our conjugate employs a non-traditional payload (M-GB), a cell-impermeable cysteine protease inhibitor that remains inactive until internalized. This design differs from conventional tubulin or DNA-damaging toxins, minimizing “bystander” toxicity and confining activity to CD74-expressing cells [19].

The characterization of this conjugate also revealed important structural insights. The peptide mapping data indicate a conjugation profile consistent with an average Drug-to-Antibody Ratio (DAR) greater than 2. While earlier-generation ADCs often targeted specific interchain cysteines, our analysis reveals that the reduction and conjugation conditions enabled modification of structural intrachain cysteines, specifically within the variable domain. Given that antibodies are homodimers, the simultaneous detection of high-intensity signals in the variable, hinge/Fc, and C-terminal regions suggests a heterogeneous population in which individual antibody molecules likely carry the payload at multiple sites concurrently. This “promiscuous” cysteine conjugation strategy maximizes drug loading; however, the modification of the variable region suggests that the antibody structure underwent sufficient reduction to expose typically buried thiols. This higher drug load may confer greater potency than a lower-DAR species, provided that modification of the antigen-binding region does not compromise target recognition.

Dual inhibition of a surface receptor and an intracellular protease also have potential implications for the tumor microenvironment. CTSL and related proteases secreted by tumor and stromal cells degrade extracellular matrix components and immune signaling molecules [12,13,14], targeted delivery of a cathepsin inhibitor could both induce tumor apoptosis and preserve the structural and immune integrity of the microenvironment. Future in vivo studies will test whether CD74–M-GB ADC treatment enhances immune effector infiltration and anti-tumor activity in DLBCL.

Nevertheless, our study has some limitations. While this study provides evidence for the mechanistic efficacy of the CD74/CTSL-targeting strategy in primary patient samples, several limitations must be addressed to avoid overstating the readiness for clinical translation. First, while our transcriptomic analysis identified a high-risk CD74^+^/CTSL^+^ signature, we acknowledge that these GEPIA2-based survival results are univariate and do not account for established clinical or molecular variables. Consequently, this signature should be interpreted as an exploratory finding that requires further validation in independent cohorts, where adjustment for factors such as the International Prognostic Index (IPI) is feasible. Consequently, our findings regarding the CD74/CTSL axis are observed across the DLBCL cohort, irrespective of TP53 mutation status, and are not limited to TP53 status. Second, our data relies on ex vivo and in vitro models. Although autologous serum culture provides a proxy for stability, full in vivo pharmacokinetic (PK) and pharmacodynamic (PD) profiling in animal models is essential to confirm that the linker withstands systemic circulation and that the conjugate achieves sufficient tumor accumulation in a 3D tissue environment. Second, the peptide mapping analysis revealed a heterogeneous conjugation profile (DAR > 2); it specifically identified modifications within the variable heavy (V_H_) domain. Modification of the antigen-binding region can theoretically impair binding affinity; however, our functional data confirms that target recognition remained sufficiently intact to drive potent and selective cytotoxicity in CD74-positive cells. While this promiscuous conjugation yields high potency, clinical development often favors more homogeneous drug substances to ensure consistent batch-to-batch pharmacokinetics. Future optimization may require site-specific conjugation technologies (such as enzymatic conjugation) to refine the DAR. Finally, while we demonstrated selectivity against antigen-negative cell lines, comprehensive in vivo toxicology studies will be required to rule out off-target uptake by healthy tissues expressing low levels of CD74 or cathepsins.

## 5. Conclusions

In conclusion, dual targeting of CD74 and CTSL represents a promising mechanistic approach for aggressive B-cell lymphomas. We have demonstrated that an ADC can be engineered to precisely deliver a cell-impermeable cathepsin inhibitor to lymphoma cells, resulting in potent tumor cell killing while sparing normal cells. This strategy uniquely addresses two critical hurdles in DLBCL treatment: surface antigen escape and intracellular resistance pathways.

The translation implications of these findings are significant. A CD74-targeted cathepsin L inhibitor ADC may provide salvage therapy for patients with refractory DLBCL or those who cannot benefit from CD20-directed therapies. By validating CTSL as a therapeutic target and introducing an ADC with a non-classical biochemical payload, our work lays the groundwork for the clinical evaluation of a first-in-class biologic.

Ultimately, this approach establishes a new framework where tumor cell surface targeting is combined with the modulation of a key tumor-promoting enzyme, thereby integrating direct tumor cytotoxicity with the alteration of the tumor microenvironment. This dual-target strategy exemplifies the next generation of precision medicine for lymphoma and warrants further investigation in advanced preclinical models to fully realize its clinical promise.

## Data Availability

All data generated or analyzed in this study are included in this published article and its Supplementary Materials.

## Supplementary Materials

The following supporting information can be downloaded at: https://www.mdpi.com/article/10.3390/cells15030291/s1, Figure S1: Impact of Antibody-Drug Conjugates on Cathepsin Activity. Cathepsin activity was assessed in cells treated with Anti-CD74 or Rituximab (RTX) based conjugates using the activity-based probe GB123; Table S1:LC-MS/MS identification of peptides modified by the M-GB inhibitor.
