# Supplementary material for: CD74-Targeted Cathepsin-Inhibitor Antibody–Drug Conjugate Triggers Apoptosis in DLBCL
Source: Cells. 2026 Feb 4;15(3):291. doi: 10.3390/cells15030291 (PMC12896589; doi:10.3390/cells15030291)
Supplement: Supplementary file 1 [file cells-15-00291-s001.zip › cells-4062872-supplementary.pdf]

Supplementary figures and tables

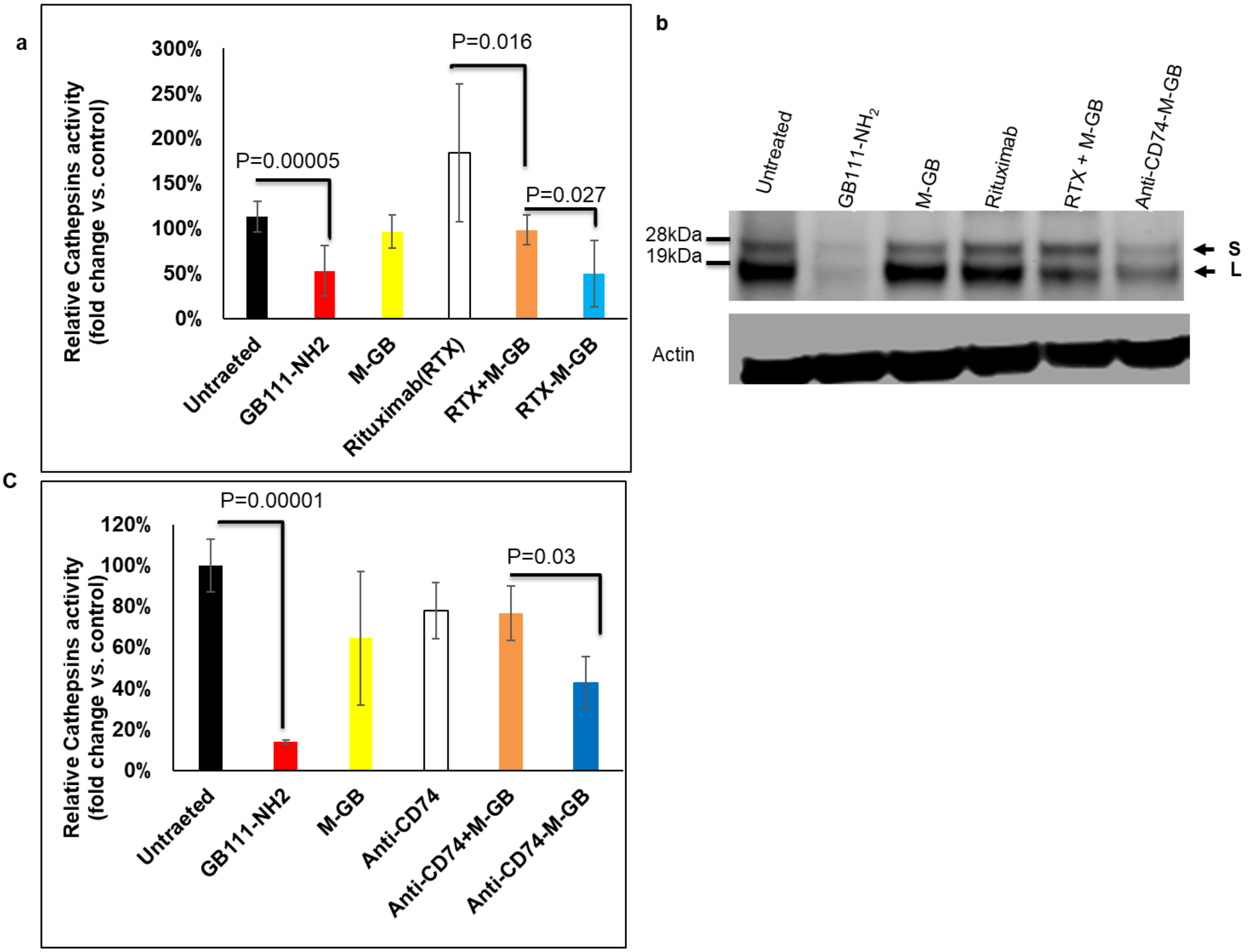

Supplementary Figure 1: Impact of Antibody-Drug Conjugates on Cathepsin Activity. Cathepsin activity was assessed in cells treated with Anti-CD74 or Rituximab (RTX) based conjugates using the activity-based probe GB123. (a) Densitometric quantification of cathepsin activity in cells treated with Rituximab (RTX) and its derivatives. Rituximab treatment alone significantly increased cathepsin activity (*P*=0.016 vs untreated), whereas the RTX-M-GB conjugate prevented this increase (*P*=0.027 vs RTX+M-GB), maintaining activity at basal levels.(b) Representative activity-based protein profiling (ABPP) gel showing cathepsin labeling (top, GB123) and beta-Actin loading control (bottom).Lysates were labeled with GB123 for 1 hour. Note the accumulation of active cathepsins in the Rituximab-treated lane (Lane 4) compared to the reduction seen with the M-GB conjugates.(c) Densitometric quantification of cathepsin activity in cells treated with Anti-CD74 antibody and its conjugates. Treatment

with Anti-CD74-M-GB significantly reduced cathepsin activity compared to controls (*P*=0.03). Statistics: All bar graphs represent the mean ± SEM of *n*=3-5 independent biological replicates. Densitometry values were normalized to beta-Actin loading controls for each respective experiment. *P*-values were determined using a student's t-test.

Supplementary Table 1.

Peptide	Unique	-10lgP	Mass	Length	ppm	m/z	z	RT	Intensity Prot1	Fraction	Scan	#Spec	#Spec Prot1	Start	End	PTM	AScore
D.TLMISRTPEVTC(+736.33)VVVDVSHEDPEVK.F	N	33.26	3518.722	25	4.6	880.6919	4	70.88	0	1	31331	1	1	254	278	GB111-NH2-Malimide-H	C12:GB111-NH2-Malimide-H:1000.00
E.VTC(+736.33)VVVDVSHEDPEVK.F	N	32.86	2490.19	16	-8	831.0639	3	71.91	5.48E+07	1	32102	1	1	263	278	GB111-NH2-Malimide-H	C3:GB111-NH2-Malimide-H:1000.00
K.SSSTAYMQLSSLTSEDSAVYYC(+736.33).A	Y	29.41	3128.343	22	12.5	783.1027	4	79.7	5.32E+07	1	38325	1	1	75	96	GB111-NH2-Malimide-H	C22:GB111-NH2-Malimide-H:1000.00
V.TC(+737.34)VVVDVSHEDPEVK.F	N	36.97	2392.129	15	8.9	599.0449	4	79.01	4.87E+07	1	37738	1	1	264	278	GB111-NH2-Malomide	C2:GB111-NH2-Malomide:1000.00
R.TPEVTC(+737.34)VVVDVSHEDPEVK.F	N	35.87	2818.341	19	7.7	564.6797	5	69.25	5.90E+07	1	30161	1	1	260	278	GB111-NH2-Malomide	C6:GB111-NH2-Malomide:1000.00
R.VEAEDAATYYC(+737.34)Q(+.98)Q(+.98)WTSNPPTFGGGTK.L	Y	44.71	3559.544	26	3.6	890.8964	4	79.21	1.65E+07	1	37904	1	1	555	580	GB111-NH2-Malomide; Deamidation (NQ)	C11:GB111-NH2-Malomide:1000.00;Q12:Deamidation (NQ):39.44;Q13:Deamidation (NQ):42.68
R.VEAEDAATYYC(+737.34)QQW(+15.99)TSNPPTFGGGTK.L	Y	27.51	3573.571	26	-12.9	1192.182	3	79.38	0	1	38051	1	1	555	580	GB111-NH2-Malomide; Oxidation (HW)	C11:GB111-NH2-Malomide:1000.00;W14:Oxidation (HW):1000.00

LC-MS/MS identification of peptides modified by the GB111-NH2 probe. List of tryptic peptides identified by liquid chromatography-tandem mass spectrometry (LC-MS/MS) containing the specific GB111-NH2-Maleimide modification. Columns indicate the peptide sequence (with the modification mass shift indicated in parentheses), uniqueness of the peptide, statistical confidence scores (-10lgP), physical properties (Mass, Length, m/z, Charge), and the specific localization of the Post-Translational Modification (PTM) to cysteine residues. The high A-scores confirm the precise localization of the GB111-NH2 adduct to the indicated cysteine sites.
